# Evolution of medical thoracoscopy in developing countries

**DOI:** 10.12669/pjms.331.12257

**Published:** 2017

**Authors:** Nasir Siddique, Tajwar Nasir

**Affiliations:** 1 Dr. Nasir Siddique, MBBS, MRCP, FCCP. Consultant in Respiratory Medicine, Kettering General Hospital, Kettering, United Kingdom; 2 Tajwar Nasir, BSc (Hons). Department of Medicine, University College London, United Kingdom

Pleural effusion constitutes around 25% of the workload in respiratory clinics yet the elucidation of underlying pathology in pleural effusion remains a diagnostic challenge. The most feared diagnosis in pleural effusion of unknown origin is malignancy, where the probability of a definitive diagnosis from aspiration, even in the most experienced hands, is approximately 60%.^1^ The dilemma, therefore, arises when considering the remaining 40%. Ideally these patients should have a pleural biopsy, either by medical thoracoscopy or video aided thoracoscopy by a thoracic surgeon. However, access to a thoracic surgeon remains a challenge in many developing countries and Pakistan is no exception.

Medical thoracoscopy is a minimally invasive technique performed by pulmonologists under local anaesthesia. It can be used to visualise the pleural space, and allows diagnostic biopsies to be performed. Furthermore, it can be used to perform therapeutic procedures, such as drainage of massive effusions and pleurodesis with high success rates.^2^ This tried and tested technique can be particularly useful in developing countries, such as Pakistan, where it can be used to treat the highly prevalent tuberculous empyema.^3^

Medical thoracoscopy has emerged as a great diagnostic technique to aid diagnosis in difficult scenarios. It is a rapidly expanding tool used in both the developed and developing world and has cemented its place in centres where there are no thoracic surgeons. In the UK, the number of chest physicians trained in this technique has risen enormously in the last decade. It has greatly facilitated the service provision, even in centres with thoracic surgeons, as the trained pulmonologist can share the workload. It is encouraging to see that larger centres in Pakistan have adopted this approach but it is still vastly under-utilised when compared with the rest of the world. This is a technique that has been adopted in many third world countries, such as Sri Lanka^4^ and India^5^, with great outcomes.

Despite enthusiasm and phenomenal interest shown from delegates in the recently held training workshop in Pakistan, there remain significant barriers to increasing national uptake of this technique. The contributing factors are poor access to training, a lack of supporting infrastructure and a lack of available funding. There has also been a palpable lack of interest shown by industry partners. The authors believe that industry partners could play a pivotal role in supporting educational activities, providing demonstrations of instrument use and raising awareness at conferences and seminars.

There are a few encouraging examples of concerted efforts being made in some hospitals: the team at a major teaching hospital in Lahore acquired the necessary infrastructure for medical thoracoscopy through purely philanthropic means and obtained training through consultant-led hands on workshops on a regular basis. The thoracoscopy service at this hospital is now successfully up and running. Perhaps a similar approach could be adopted in other centres wishing to embark upon such a venture. However, in order for long-term sustainability, a government led initiative is necessary to make these services widely available.

We believe that this relatively novel and inexpensive technique has a great future in developing countries in the diagnosis of pleural effusion of unknown origin. With increasing awareness and adoption of this technique, it is envisaged that most district and teaching hospitals should be able to provide such service. The evidence suggests that this is safe,^6^ well tolerated and provides a high diagnostic yield.^7^ It also shortens hospital stay from ten days using the conventional approach down to four days,^8^-^10^ thus cutting healthcare costs and enhancing quality of life in patients with limited life expectancy. Thus, medical thoracoscopy can be a revolutionary approach in countries severely constrained in health budgets.

In summary, the key steps necessary for the success of this program remain the standardisation of this technique, inclusion within the curriculum for pulmonology training, and regular expert led workshops.
